# Forecasting the spread of COVID-19 based on policy, vaccination, and Omicron data

**DOI:** 10.1038/s41598-024-58835-9

**Published:** 2024-04-30

**Authors:** Kyulhee Han, Bogyeom Lee, Doeun Lee, Gyujin Heo, Jooha Oh, Seoyoung Lee, Catherine Apio, Taesung Park

**Affiliations:** 1 Interdisciplinary Program of Bioinformatics, Seoul National University, Seoul, Republic of Korea; 2 Department of Industrial Engineering, Seoul National University, Seoul, Republic of Korea; 3 Ross School of Business, University of Michigan-Ann Arbor, Ann Arbor, MI, United States; 4 College of Humanities, Seoul National University, Seoul, Republic of Korea

**Keywords:** Public health, Epidemiology

## Abstract

The COVID-19 pandemic caused by the novel SARS-COV-2 virus poses a great risk to the world. During the COVID-19 pandemic, observing and forecasting several important indicators of the epidemic (like new confirmed cases, new cases in intensive care unit, and new deaths for each day) helped prepare the appropriate response (e.g., creating additional intensive care unit beds, and implementing strict interventions). Various predictive models and predictor variables have been used to forecast these indicators. However, the impact of prediction models and predictor variables on forecasting performance has not been systematically well analyzed. Here, we compared the forecasting performance using a linear mixed model in terms of prediction models (mathematical, statistical, and AI/machine learning models) and predictor variables (vaccination rate, stringency index, and Omicron variant rate) for seven selected countries with the highest vaccination rates. We decided on our best models based on the Bayesian Information Criterion (BIC) and analyzed the significance of each predictor. Simple models were preferred. The selection of the best prediction models and the use of Omicron variant rate were considered essential in improving prediction accuracies. For the test data period before Omicron variant emergence, the selection of the best models was the most significant factor in improving prediction accuracy. For the test period after Omicron emergence, Omicron variant rate use was considered essential in deciding forecasting accuracy. For prediction models, ARIMA, lightGBM, and TSGLM generally performed well in both test periods. Linear mixed models with country as a random effect has proven that the choice of prediction models and the use of Omicron data was significant in determining forecasting accuracies for the highly vaccinated countries. Relatively simple models, fit with either prediction model or Omicron data, produced best results in enhancing forecasting accuracies with test data.

## Introduction

Coronavirus Disease 19 (COVID-19) is a severe respiratory syndrome caused by 2019-nCoV virus^1^. Since the first case in Wuhan, China, in 2019, there have been 540,625,513 confirmed cases and 6,331,685 deaths worldwide (June 13, 2022)^2^. In the early stages of the epidemic, most countries applied intensive restriction policies including lockdowns to control the spread of COVID-19, since neither vaccines nor cures had yet been developed^3^. After vaccines’ development, some countries including Israel, the United Kingdom, and Singapore increased their vaccination rates (VRs) rapidly to ease restrictions^4–6^. Currently, most countries are administering booster doses of vaccination to their fully vaccinated population. Despite these efforts, the emergence of new SARS-CoV-2 virus variants including Omicron (whose first specimen was collected on November 9, 2021)^7^ and the resulting risk of a breakthrough infection^8^ showed that humans could not yet fully escape from the COVID-19 pandemic. In particular, Omicron variants are known to have a higher basic reproduction number (R0) of up to 10 compared to 2.5 of the original SARS-CoV-2 series or below 7 for Delta variants^9^. This high reproduction number directly affects the rate of spread, which in turn, can be observed by the number of confirmed cases. Thus, it is essential to provide separate predictions for each using data before and after Omicron variant emergence. Policy and vaccination data may play an important role in forecasting the spread of the pandemic before Omicron appearance, whereas Omicron rate (OR) data itself may be considered pivotal for prediction after its appearance.

During the COVID-19 epidemic, predicting the trend of the epidemic can help to allocate healthcare services efficiently and measure the impact of intervention policies^10^. There are several important indicators of an epidemic such as (1) new confirmed cases, (2) new deaths, and (3) Intensive Care Unit (ICU) patients. These indicators could be predicted by several prediction models and predictor variables.

For prediction models, (1) mathematical models, (2) statistical models, and (3) AI/machine learning models can be used. For mathematical models, the Susceptible-Exposed-Infectious-Quarantined-Recovered (SEIQR) model was applied to predict daily confirmed cases, isolated people, and peak date of isolation during the first outbreak in Daegu, South Korea^11^. Using the number of cumulative cases, suspected, recovery, deaths, and quarantined population in mainland China, the Susceptible-Exposed-Infectious-Quarantined-Diagnosis-Recovered (SEIQDR) model was used to predict cumulative future cases, infection rate, and regeneration number of the disease^12^. However, both models were not able to cover the effects of vaccinations and the insusceptible group with immunity. Thus, we developed and applied our SEIQRDV3P model, which is a modification of the Susceptible-Exposed-Infectious-Quarantined-Recovered-Deceased-Vaccinated-Unprotected-Protected (SEIQRDVUP) model.

For statistical models, Auto-Regressive Integrated Moving Average (ARIMA) models, single and double exponential methods, and trend models were used for the prediction of confirmed cases in India. Among them, ARIMA models showed the best performance^13^. However, seasonality and regression were not considered in this study, limiting the most appropriate time series model to a relatively simple ARIMA model (2,2,2). In the case of a study regarding the prediction of ICU patients in Berlin, Lombardy, and Madrid, hyperparameters including appropriate time lags, time in ICUs, and ICU rates were first determined. Then simple linear and exponential models were applied to predict the number of future ICU patients^14^. The above models utilized relatively short-term data usually from one month to three months at the longest, both for training and prediction. Conversely, considering that the pattern of patients has changed dramatically with various outbreaks, we applied a long training period (14 months) to better observe the overall trend.

Most of these statistical approaches are only valid for short-term forecasting. Meanwhile, for long-term and more accurate predictions, AI/machine learning models were considered. Two hybrid methods of Artificial Neural Network (ANN) algorithms such as the Multi-Layered Perceptron-Imperialist Competitive Algorithm (MLP-ICA) and the Adaptive Neuro-Fuzzy Inference System (ANFIS) were used for a 6-month future prediction of the number of cases and mortality in Hungary^15^. In addition, nonlinear autoregressive artificial neural network (NARANN)^16^, Long Short-Term Memory (LSTM)^17^, and ANFIS with Chaotic Marine Predators Algorithm (CMPA)^18^ were also used to predict the spread of COVID-19. Most of these AI/machine-learning models were shown to have better performance than statistical models. However, no single model performs best across different countries, due to their diverse pandemic trends^19^. Recently, AI/machine learning models have also been used in other various prediction problems during the COVID-19 epidemic, such as detecting COVID-19 infections from CT/X-ray images using CNNs^20^ and identifying virus subsequences based on infection propagation mechanisms using the Fragmented Local Aligner Technique (FLAT) model^21^.

Some prediction models can also use additional predictor variables to improve the accuracy of prediction. Several predictor variables include (1) previously new confirmed patients/critical patients / new deaths at specific periods (ex: before 7 days), (2) VR, (3) daily restriction policy, (4) mobility, etc. For restriction policies, one study has considered lockdown variables and population demographics of eight European countries. It showed that stringency indices (SI), including lockdown, have a large effect on both infection and mortality rates^22^. In another study using mobility data to predict confirmed cases in Spain, an explanatory variable was first obtained by an ensemble of time series models and machine learning models. Then a multivariate regression model using that explanatory variable and Google mobility variables of lags 1 to 7 was introduced for forecasting cumulative cases^23^. However, there exists little research involving VRs and their lagged data as predictor variables for forecasting future cases.

Therefore, in this paper, we tried to compare the accuracy of predictions when using various prediction models and predictor variables. First, we predicted the newly confirmed patients, ICU patients, and new deaths in seven countries namely, Denmark, Israel, Japan, Singapore, South Korea, the UK, and the USA. We selected them from developed countries with high VRs (higher than 70% fully vaccinated, as of December 31st, 2021). In addition, we tried to show the effect of Omicron emergence by analyzing both periods, before Omicron appearance and after Omicron appearance. We used the last 7 days for each period as test data, and the rest were used for training the model. We chose three statistical models (Generalized Additive Model (GAM), Time Series Generalized Linear Model (TSGLM), and Multiplicative Seasonal ARIMA), two AI/machine learning algorithms (lightGBM and Bi-directional Long Short-Term Memory (Bi-LSTM)), and one mathematical model (SEIQRDV3P) to forecast the spread of COVID-19. These six prediction models and three predictor variables were used for prediction.

The multiplicative seasonal ARIMA model is a fundamental prediction model for time series data. TSGLM is another representative method for analyzing data when observations depend on past data. GAM deals with the nonlinear relationship between predictor variables and response variables. Among various AI/machine learning models, lightGBM with its fast learning time, and Bi-LSTM which learns time series data bidirectionally, were chosen. Lastly, the SEIQRDV3P model was developed to handle three different VRs depending on the number of inoculations. We also used various predictor variables (VR, SI, OR) to increase the forecasting accuracy. Especially, we used VRs depending on the different number of inoculations, from the first dose, the second dose (fully vaccinated), and the third dose (also called the booster shot). Secondly, we compared the accuracy of each prediction.

A Linear Mixed Model (LMM)^24^ was used to investigate the impact of predictive models and predictor variables on prediction accuracy across different countries. Bayesian Information Criterion (BIC)^25^ was applied for selecting the best model. Prediction models and Omicron variants were significant in the prediction of test data.

In short, the major contributions of our study are as follows. First, we utilized various types of prediction models and predictor variables including vaccination coefficients for forecasting different indicators of COVID-19. Secondly, we introduced an approach using forecast error as a response variable for LMMs, measuring the impact of each predictor and making our results generalizable to other countries for further research. Finally, we have discovered the choice of the prediction model and the use of OR are significant in improving forecasting accuracy for each country.

## Results

### Fitting and choosing the best LMMs

We have used LMMs to distinguish predictor variables with the largest influence on forecasting accuracy. As we are interested in the distinct effects of prediction models, vaccination coefficients, and Omicron variant on confirmed cases, deaths, and ICU patients, these were considered fixed effects, whereas countries were treated as random effects. All variables are categorical and dummy coded, including interaction terms, but for convenience, we suggested two models. (LMM a) and (LMM b) are the full models for test periods #1 (before Omicron) and #2 (after Omicron), respectively. Compared to (LMM b), Omicron predictor variable and interaction terms involving Omicron variant data are not included as response variables in (LMM a).

LMM a $${y}_{ijl}={\beta }_{0}+{\beta }_{\mathrm{1,1}}{a}_{i,1}+\dots +{\beta }_{\mathrm{1,5}}{a}_{i,5}+{\beta }_{\mathrm{2,1}}{b}_{j,1}+\dots +{\beta }_{\mathrm{2,4}}{b}_{j,4}+{\gamma }_{1}{d}_{ij}+{\delta }_{l}+{\varepsilon }_{ijl}$$

LMM b $${y}_{ijkl}={\beta }_{0}+{\beta }_{\mathrm{1,1}}{a}_{i,1}+\dots +{\beta }_{\mathrm{1,5}}{a}_{i,5}+{\beta }_{\mathrm{2,1}}{b}_{j,1}+\dots +{\beta }_{\mathrm{2,4}}{b}_{j,4}+{\beta }_{3}{c}_{k}+{\gamma }_{1}{d}_{ij}+{\gamma }_{2}{e}_{jk}+{\gamma }_{3}{f}_{ki}+{\gamma }_{4}{g}_{ijk}+{\delta }_{l}+{\varepsilon }_{ijkl}$$

${a}_{i,v}$ and ${b}_{j,v}$ refer to dummy coded prediction models and vaccination coefficients, respectively. ARIMA prediction model without vaccination coefficient and Omicron variant usage for Denmark serves as the baseline model. ${d}_{ij}$ refers to the interaction between prediction models and vaccination coefficients. ${e}_{jk}$ refers to the interaction between vaccination coefficients and the use of Omicron variants. ${f}_{ki}$ refers to the interaction between the use of Omicron variants and prediction models. ${g}_{ijk}$ refers to the three-way interaction between prediction models, vaccination coefficients, and the use of Omicron variants. ${\delta }_{l}$ refers to country, or our random intercept effect. ${\varepsilon }_{ijkl}$ and ${y}_{ijkl}$ refer to the error term and log_10_WMAPE value for each prediction model, vaccination coefficient, use of Omicron variants, and country, respectively. WMAPE values were log-transformed to reduce the influence of outliers and to better follow a normal distribution. We assumed that ${\delta }_{l}$ and ${\varepsilon }_{ijkl}$ follow normal distributions N (0, ${{\sigma }_{l}}^{2}$) and N(0, ${\sigma }^{2}$), respectively. Note that the extended SEIR model is not designed to use Omicron variants as its variable, thus, we do not fit ${y}_{4j1l}$ values. Table 1 shows coefficient values for our model.

**Table 1:** Coefficient values for linear mixed effect model (test period #2).

***v***	${a}_{i,v}$(Prediction Model)	${b}_{j,v}$(Vaccination Coefficient)	${c}_{k}$(Omicron variant usage)	${\delta }_{l}$(Country)
Baseline	${a}_{i,1}=\cdot \cdot \cdot ={a}_{i,5}=0$(ARIMA)	${b}_{j,1}=\cdot \cdot \cdot ={b}_{j,4}=0$ (Vaccination coefficient unused)	${c}_{k}$= 0 (Omicron variant unused)	*l* = 0 (DNK)
1	${a}_{i,1}=1$(BiLSTM), 0 (otherwise)	${b}_{j,1}$= 1 (1^st^-vaccination), 0 (otherwise)	${c}_{k}$= 1 (Omicron variant used)	*l* = 1 (GBR)
2	${a}_{i,2}=1$(GAM), 0 (otherwise)	${b}_{j,2}$= 1 (2^nd^-vaccination), 0 (otherwise)		*l* = 2 (ISR)
3	${a}_{i,3}=1$(lightGBM), 0 (otherwise)	${b}_{j,3}$= 1 (3^rd^-vaccination), 0 (otherwise)		*l* = 3 (JPN)
4	${a}_{i,4}=1$(SEIR), 0 (otherwise)	${b}_{j,4}$= 1 (1st, 2nd, 3rd vaccinations), 0 (otherwise)		*l* = 4 (KOR)
5	${a}_{i,5}=1$(TSGLM), 0 (otherwise)			*l* = 5 (SGP)
6				*l* = 6 (USA)

For test period #1, we do not have ${c}_{k}$ predictor variables. Interaction coefficients are not shown. Country codes are as follows.

*DNK* Denmark, *GBR* Great Britain, *ISR* Israel, *JPN* Japan, *KOR* South Korea, *SGP* Singapore, *USA* United States of America.

Table 2 and Table 3 show our data structure with predictor variables and response variables for test period #1 (before Omicron) and #2 (after Omicron), respectively.

**Table 2:** Overall data structure for test period #1.

Country	Vaccination coefficient	Prediction model	*y*_*ijl*_
DNK (*l* = 0)	Unused (${b}_{j,1}=\cdot \cdot \cdot ={b}_{j,4}=0$)	ARIMA (${a}_{i,1}=\cdot \cdot \cdot ={a}_{i,5}=0$)	*y*_000_
		BiLSTM (${a}_{i,1}=1$)	*y*_100_
		GAM (${a}_{i,2}=1$)	*y*_200_
		lightGBM (${a}_{i,3}=1$)	*y*_300_
		SEIR (${a}_{i,4}=1$)	*y*_400_
		TSGLM (${a}_{i,5}=1$)	*y*_500_
	1st-vaccination (${b}_{j,1}$ = 1)	ARIMA (${a}_{i,1}=\cdot \cdot \cdot ={a}_{i,5}=0$)	*y*_010_
		$\cdot \cdot \cdot$	$\cdot \cdot \cdot$
		TSGLM (${a}_{i,5}=1$)	*y*_510_
	$\cdot \cdot \cdot$	$\cdot \cdot \cdot$	$\cdot \cdot \cdot$
	1st, 2nd, 3rd vaccinations (${b}_{j,4}$ = 1)	TSGLM (${a}_{i,5}=1$)	*y*_540_
$\cdot \cdot \cdot$	$\cdot \cdot \cdot$	$\cdot \cdot \cdot$	$\cdot \cdot \cdot$
USA (*l* = 6)	1st, 2nd, 3rd vaccinations (${b}_{j,4}$ = 1)	TSGLM (${a}_{i,5}=1$)	*y*_546_

**Table 3:** Overall data structure for test period #2.

Country	Omicron variant usage	Vaccination coefficient	Prediction model	*y*_*ijkl*_
DNK (*l* = 0)	Unused (${c}_{k}$ = 0)	Unused (${b}_{j,1}=\cdot \cdot \cdot ={b}_{j,4}=0$)	ARIMA (${a}_{i,1}=\cdot \cdot \cdot ={a}_{i,5}=0$)	*y*_0000_
			BiLSTM (${a}_{i,1}=1$)	*y*_1000_
			GAM (${a}_{i,2}=1$)	*y*_2000_
			lightGBM (${a}_{i,3}=1$)	*y*_3000_
			SEIR (${a}_{i,4}=1$)	*y*_4000_
			TSGLM (${a}_{i,5}=1$)	*y*_5000_
		1st-vaccination (${b}_{j,1}$ = 1)	ARIMA (${a}_{i,1}=\cdot \cdot \cdot ={a}_{i,5}=0$)	*y*_0100_
			$\cdot \cdot \cdot$	$\cdot \cdot \cdot$
			TSGLM (${a}_{i,5}=1$)	*y*_5100_
		$\cdot \cdot \cdot$	$\cdot \cdot \cdot$	$\cdot \cdot \cdot$
		1st, 2nd, 3rd vaccinations (${b}_{j,4}$ = 1)	TSGLM (${a}_{i,5}=1$)	*y*_5400_
	Used (${c}_{k}$ = 1)	Unused (${b}_{j,1}=\cdot \cdot \cdot ={b}_{j,4}=0$)	ARIMA (${a}_{i,1}=\cdot \cdot \cdot ={a}_{i,5}=0$)	*y*_0010_
		$\cdot \cdot \cdot$	$\cdot \cdot \cdot$	$\cdot \cdot \cdot$
		1st, 2nd, 3rd vaccinations (${b}_{j,4}$ = 1)	TSGLM (${a}_{i,5}=1$)	*y*_5410_
$\cdot \cdot \cdot$	$\cdot \cdot \cdot$	$\cdot \cdot \cdot$	$\cdot \cdot \cdot$	$\cdot \cdot \cdot$
USA (*l* = 6)	Used (${c}_{k}$ = 1)	1st, 2nd, 3rd vaccinations (${b}_{j,4}$ = 1)	TSGLM (${a}_{i,5}=1$)	*y*_5416_

We created all possible combinations of LMMs using fixed effects and their interaction terms. Note that the number of parameters slightly differs since our extended SEIR model does not provide predictions for ICU patients due to model limitations. For convenience, we denote our prediction model parameter as *model*, vaccination coefficient parameter as *cov*, usage of Omicron variant parameter as *omicron*, country parameter as a *country*, and log_10_WMAPE forecast as *y*. Our model formulae refer to which are used in the R *lmerTest* package. For instance, the formula *y* ~ *omicron* + (1|*country*) refers to the reduced model.

$$y_{ijkl} = \beta_{0} + \beta_{3} c_{k} + \delta_{l} + \varepsilon_{ijkl}$$

Among those, we selected the best models based on AIC and BIC values for each of the 6 cases; raw confirmed cases, smoothed confirmed cases, raw daily deaths, smoothed daily deaths, raw ICU patients, and smoothed ICU patients. For test period #1, regardless of prediction case and criterion (AIC or BIC), the best model was *y* ~ *model* + (1 | *country*). Table 4 lists the best models for each case in test period #2. Note that the best models selected based on AIC were more complex than those based on BIC since in our study we have a total of 210 observations per country, i.e., log(*n*) = 5.35 in (26) and (27).

**Table 4:** The best model for each prediction case based on AIC and BIC (test period #2).

Criterion	Prediction case		Best model	Number of parameters
AIC	Confirmed cases	Raw	*y* ~ *model* * *cov* + *cov* * *omicron* + *omicron* * *model* + *model* * *cov* * *omicron* + (1 | *country*)	57
		Smoothed	*y* ~ *omicron* + (1|*country*)	4
	Daily deaths	Raw	*y* ~ *omicron* + (1|*country*)	4
		Smoothed	*y* ~ *model* + (1 | *country*)	8
	ICU patients	Raw	*y* ~ *model* * *cov* + (1 | *country*)	27
		Smoothed	*y* ~ *model* * *cov* + *omicron* + (1 | *country*)	28
BIC	Confirmed cases	Raw	*y* ~ *omicron* + (1|*country*)	4
		Smoothed	*y* ~ *omicron* + (1|*country*)	4
	Daily deaths	Raw	*y* ~ *omicron* + (1|*country*)	4
		Smoothed	*y* ~ *model* + (1 | *country*)	8
	ICU patients	Raw	*y* ~ *model* + (1 | *country*)	7
		Smoothed	*y* ~ *model* + (1 | *country*)	7

When selecting the best model for each prediction case, the RMSE values for the best models were calculated. For smoothed confirmed cases, raw daily deaths, and smoothed daily deaths, the same models were selected by AIC and BIC. Their RMSE were 0.6182, 104.9059, and 1.6723, respectively. For raw confirmed cases, the RMSE for the BIC best model was 10.6081, slightly higher than 7.5671, the RMSE for the AIC best model. For raw ICU patients, RMSE for the BIC best model was 0.1196, compared to 0.1037 for the AIC best model. Lastly, for smoothed ICU patients, RMSE for the BIC best model was 0.0427, compared to 0.0384 for the AIC best model. Since BIC tends to limit the number of parameters, it is natural that more complex models selected by AIC have lower RMSE values. However, if there is not much difference in RMSE between AIC and BIC best models, the principle of parsimony can be applied and BIC can be a better criterion.

Supplementary Figs S1-S4 are the visualizations of all AIC and BIC values for each test period and each prediction case, respectively. In Figs S3 and S4, we can observe that the best model selection is more consistent when BIC is used. Thus, we decide to use BIC as our measure for test period #2.

### Hypothesis testing for best models

**Figure 1:**
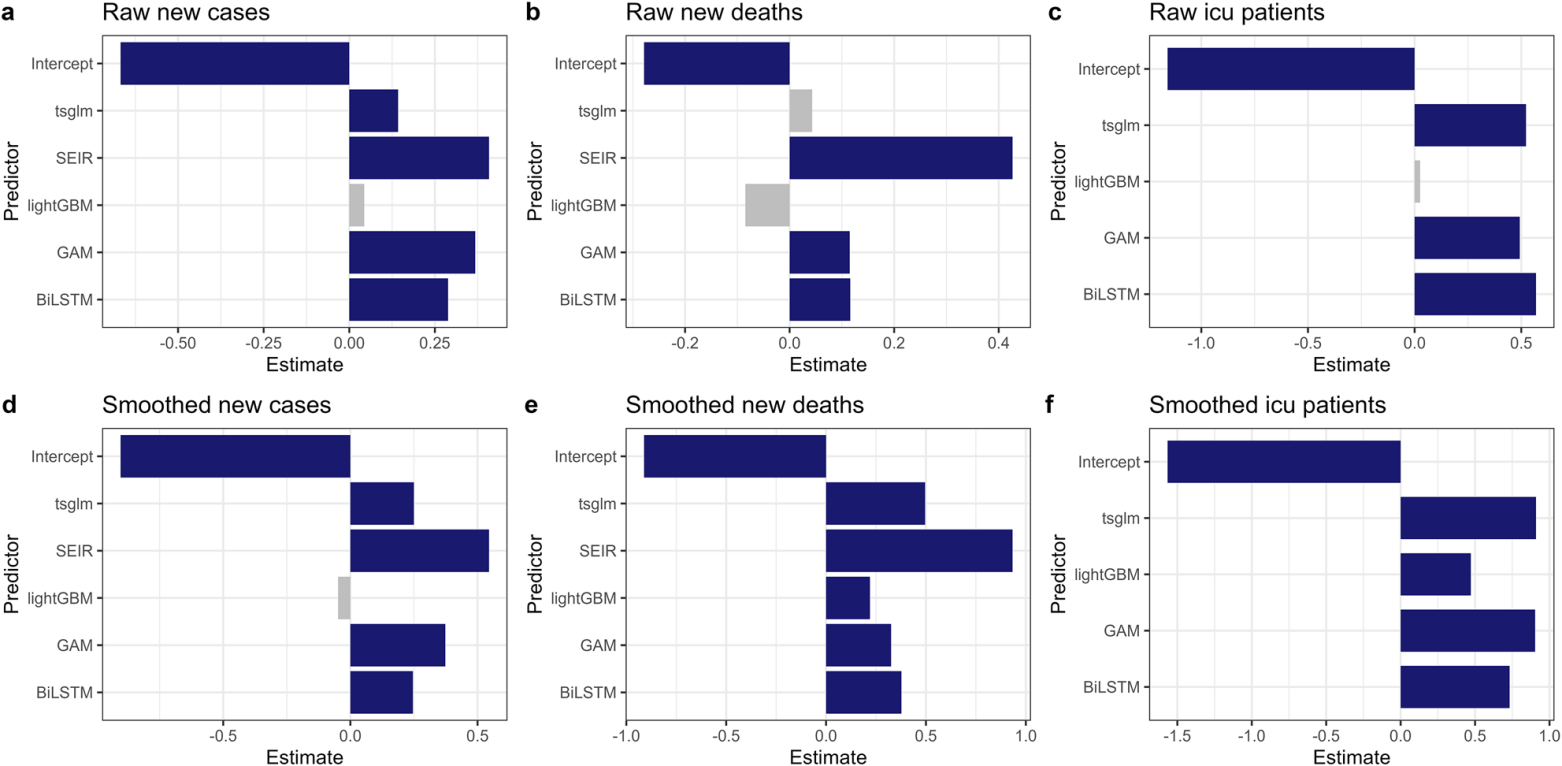
Visualization of estimate of predictors (test period #1). The blue/gray bar represents whether the corresponding predictor is significant/not significant under significance level 0.05, respectively.

**Figure 2:**
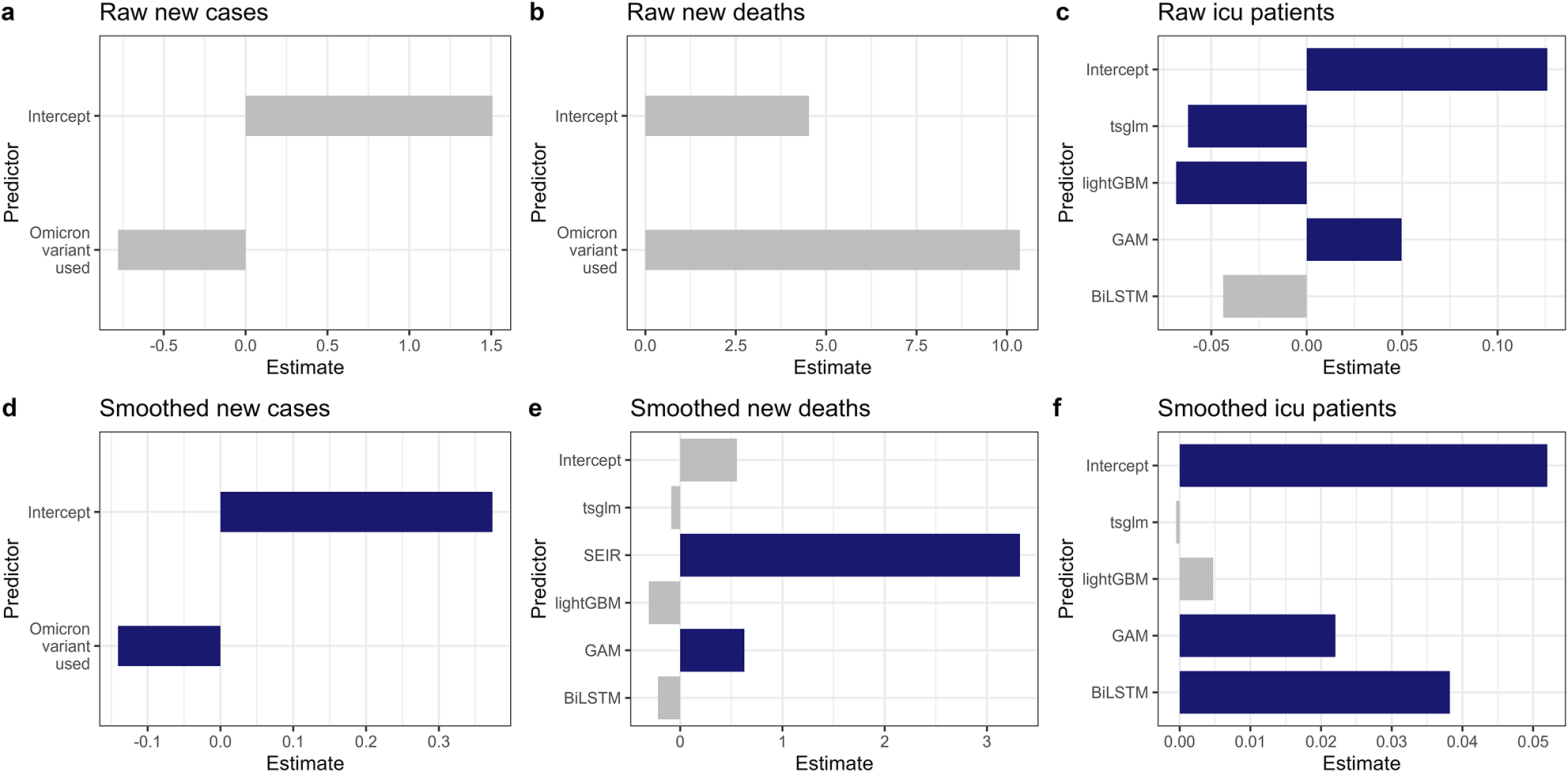
Visualization of the estimate of predictors (test period #2). The blue/gray bar represents whether the corresponding predictor is significant/not significant under significance level 0.05, respectively.

Using p-values obtained by the best models selected based on BIC measure for each prediction case, we performed the Wald test on the fixed effects and their interactions to see if each of them is significant. Figures 1 and 2 are the visualizations of the estimate of predictors in each of our best models in test periods #1 and #2, respectively.

### Hypothesis testing for test period #1

Since we have the same best model *y* ~ *model* + (1 | *country*) regardless of prediction case and criterion for test period #1, we summarize our results in a single table. Table 5 shows our results for test period #1. Our hypothesis tested are given as:

3 $$H_{0} :\beta_{1,i} \, = \,0 \, vs \, H_{a} :\beta_{1,i} \, \ne \,0 \, \left( {i\, = \,1, \, 2, \, 3, \, 4, \, 5} \right)$$

**Table 5:** Estimates and p-values for each predictor, test period #1.

Predictor (Model)	New cases				Deaths				ICU patients			
	Raw		Smoothed		Raw		Smoothed		Raw		Smoothed	
	Estimate	Pr( >|t|)	Estimate	Pr( >|t|)	Estimate	Pr( >|t|)	Estimate	Pr( >|t|)	Estimate	Pr( >|t|)	Estimate	Pr( >|t|)
Intercept	− 0.66790	3.50e− 05*	− 0.90356	6.04e− 05*	− 0.27847	0.0374*	− 0.91120	1.64e-05*	− 1.15828	1.32e-06*	− 1.56572	1.46e− 09*
BiLSTM	0.28847	9.70e− 06*	0.24581	0.01585*	0.11603	0.0402*	0.37717	0.000110*	0.56921	2.70e-07*	0.73186	1.31e− 11*
GAM	0.36787	1.10e− 08*	0.37328	0.00021*	0.11500	0.0361*	0.32542	0.000677*	0.49300	7.68e-06*	0.90331	1.89e− 15*
lightGBM	0.04372	0.4724	− 0.04826	0.61800	− 0.08473	0.1164	0.21973	0.017149*	0.02571	0.806	0.47151	4.12e− 06*
Extended SEIR	0.40807	1.99e− 10*	0.54506	5.96e− 08*	0.42644	1.64e-13*	0.93339	< 2e-16*	–	–	–	–
TSGLM	0.14249	0.0208*	0.24966	0.01104*	0.04311	0.4264	0.49576	2.10e-07*	0.52226	1.95e-06*	0.90945	8.25e-16*

ARIMA model is given as the baseline model. The asterisk ^*^ indicates that we can reject the null hypothesis at a significance level = 0.05.

First, we can observe that the estimate of almost all prediction models (except for lightGBM model in smoothed new cases and raw new deaths) is positive, indicating that our log WMAPE value has increased due to these models. In contrast, the baseline model, ARIMA, provides the best predictions in most cases. Furthermore, the prediction accuracy of ARIMA significantly differs from other models.

### Hypothesis testing for test period #2

The best model for raw new cases is the simplest model *y* ~ *omicron* + (1|*country*)). Thus, we tested the following hypothesis:

4 $$H_{0} :\beta_{3} \, = \,0 \, vs \, H_{a} :\beta_{3} \, \ne \,0$$

The estimates for the intercept and Omicron variant predictor are 1.5078 and -0.7793, respectively. However, their p-values are 0.0746 and 0.5011, thus we cannot reject our null hypothesis under significance level 0.05, i.e., there is no significant effect of Omicron variant usage on the prediction of raw new cases. Nevertheless, the estimated value for the Omicron variant predictor is still negative, indicating that using Omicron data contributes to lowering the mean WMAPE or improving the accuracy of the test data in general.

The best model for smoothed new cases is the simplest model *y* ~ *omicron* + (1|*country*)). Our hypothesis is the same as (H2). The estimates for the intercept and Omicron variant predictor are 0.37357 and -0.14064, respectively. In addition, their p-values are 8.53e-07 and 0.0315, thus we can reject our null hypothesis under significance level 0.05, indicating that the Omicron variant plays a significant role in the prediction of smoothed confirmed cases. Furthermore, we may conclude that using omicron data contributes to improving test data accuracy since our estimate is negative, as in 3.4.1.

The best model for raw new deaths is the simplest model *y* ~ *omicron* + (1|*country*)). Our hypothesis is the same as (H2). The estimates for the intercept and Omicron variant predictor are 4.523 and 10.356, respectively, with p-values of 0.543 and 0.351. Thus, there is no significant difference between models with and without Omicron variant usage in the prediction of raw deaths.

The best model for smoothed new deaths is the model *y* ~ *model* + (1|*country*). Our hypotheses are the same as (H1). Results are shown in Table 6. We can reject our null hypothesis under a significance level of 0.05 for two prediction models (GAM and Extended SEIR), indicating that these two models have a significant difference compared to the baseline (ARIMA) model. We also observed that GAM and Extended SEIR models are less appropriate in predicting smoothed death cases than other prediction models, as their estimates are positive. The choice of prediction model plays a significant role in the prediction of smoothed confirmed cases.

**Table 6:** Estimates and p-values for each predictor, smoothed deaths, raw ICU patients, and smoothed ICU patients.

Predictor	New deaths (Smoothed)		ICU patients (Raw)		ICU patients (Smoothed)	
	Estimate	Pr( >|t|)	Estimate	Pr( >|t|)	Estimate	Pr( >|t|)
Intercept	0.55468	0.0624	0.12606	0.00006*	0.05201	0.00222*
BiLSTM	− 0.21731	0.4695	− 0.04375	0.07106	0.03822	0.00001*
GAM	0.62766	0.0347*	0.04969	0.04752*	0.02203	0.01373*
lightGBM	− 0.30701	0.2857	− 0.06841	0.00497*	0.00473	0.58436
Extended SEIR	3.32317	< 2e− 16*	–	–	–	–
TSGLM	− 0.08712	0.7633	− 0.06217	0.01058*	− 0.0005	0.95378

The asterisk ^*^ indicates that we can reject the null hypothesis at a significance level = 0.05.

The best model for raw ICU patients is the model *y* ~ *model* + (1 | *country*). Our hypotheses are the same as (H1). Note there are no results for the extended SEIR model since it is not designed to predict ICU patients (both raw and smoothed). Results are also shown in Table 6. We can reject our null hypothesis under a significance level of 0.05 for all models except BiLSTM. This result further elaborates the choice of model is significant in deciding prediction accuracy and models except GAM performed better than the baseline model.

The best model for smoothed ICU patients is the model *y* ~ *model* + (1 | *country*). Our hypotheses are the same as (H1). Results are also shown in Table 6. We can reject our null hypothesis under a significance level of 0.05 for BiLSTM and GAM models. For smoothed ICU patients, the baseline model (ARIMA) and TSGLM showed better results than other prediction models.

## Discussions

For predictions before Omicron appearance, the ARIMA model shows consistently high prediction accuracies. ARIMA models are still constantly being used as baselines for time series prediction since seasonality can be easily applied to them and they best reflect the fact that the number of daily cases is greatly affected by the number of cases the day before than any other model. In addition, as lightGBM uses leafwise tree growth as its boosting algorithm and continuously updates its tree structure, its prediction performance is decent in most cases. In addition, since parameter tuning is more efficient in lightGBM when the number of parameters is small, test accuracies before Omicron are better than those after Omicron. For predictions after Omicron appearance, using Omicron data turned out to improve test data accuracy. This is not only because Omicron data has a weekly seasonality but also because the Omicron variant is starting to dominate over other variants. In this sense, as Omicron variants are highly infectious but not so fatal, they are selected as predictors for best models in predicting new cases but not in predicting ICU patients. For prediction models, the ARIMA model, lightGBM, and TSGLM performed fair overall. In addition to the aforementioned two, TSGLM, as a time series model which includes previous conditional means to its prediction process, provides reasonable predictions for both deaths and ICU patients.

Here, we analyzed the effects of various predictor variables and prediction models on forecasting performance. To compare our work with existing methods, we conducted a literature review investigating prediction models and predictor variables used in published COVID-19 prediction papers. Table 7 shows the summarized results of previous studies focusing on AI/machine learning models. Some of the studies used past observation to predict the response variable. For instance, some studies forecasted cumulative confirmed cases^29^ or cumulative vaccination rates^30^ using past observations. Other studies used predictor variables including temperature, mobility, and social distancing^26,27^. However, these studies only compare the prediction models, not predictor variables^26–28^. Differing from previous studies, SI, VR, and OR were used as predictor variables. These variables were rarely considered in forecasting studies, despite their evidence that they are related to the epidemic trend^31–33^. In addition, to the best of our knowledge no study has simultaneously analyzed both the effects of prediction models and predictor variables across multiple regions. In summary, our analysis has novelty compared to previous studies by employing distinct predictor variables and analyzing both prediction models and predictor variables at the same time.

**Table 7:** AI/machine learning models for forecasting the COVID-19 epidemic.

Authors	Target Regions	Response variables	Predictor variables	Prediction models
Pinter et al. (2020)	Single region	Daily new cases Mortality rate	Single variable (past observation)	Multiple models (MLP-ICA, ANFIS)
Saba and Elsheikh (2020)	Single region	Cumulative cases	Single variable (past observation)	Multiple models (ARIMA, NARANN)
Ramazi et al. (2021)	Single region	Daily new cases Daily new deaths	Multiple variables (daily COVID-19 tests daily temperature daily precipitation Google mobility)	Multiple models (LaFoPaFo,UCLA-SuEIR, STH-3PU, etc.)
Gomez-Cravioto et al. (2021)	Single region	Daily new cases Daily new deaths	Multiple variables (weather information—temperature, UV index, humidity, etc Google mobility)	Multiple models (logistic growth curve, ARIMA, LSTM, etc.)
Elsheikh et al. (2021)	Multiple regions	Cumulative cases Cumulative recovered Cumulative deaths	Single variable (past observation)	Multiple models (ARIMA, NARANN, LSTM)
Al-qaness et al. (2021)	Multiple regions	Daily new cases	Single variable (past observation)	Multiple models (ANFIS, ANFIS-MPA, ANFIS-CMPA)
Meakin et al. (2022)	Single region	Daily hospitalization	Single variable (Daily new cases with time lag)	Multiple models (baseline, Timeseries ensemble, ARIMA, etc.)

While many previous studies focused on improving prediction accuracies of daily confirmed cases, we have measured the impact of different predictors including vaccinations, policies, and prediction models on accuracies of not only confirmed cases but also deaths and ICU patients. In this regard, this general approach using linear mixed models may be applied to other pandemics in the future and presents a guideline on responding to fast-spreading diseases for various countries. Because we utilized LMMs considering the choice of a country as a random effect, we believe our result can be applied well to highly vaccinated countries. However, since our results were obtained using data from only 7 countries, the generalizability needs to be verified with more countries. Meanwhile, although VR is not a significant predictor in our results for highly vaccinated countries, it can still be considered as a candidate predictor for improving forecasting accuracy for future analysis of countries where only a small number of their population has been vaccinated.

The LMM framework we developed is practical in that it identifies which factors are significant for the accurate prediction of infectious diseases. In this study, the impact of different prediction models (mathematical, statistical, and AI/machine learning models) and predictor variables (SI, VR, OR) on forecasting performance was analyzed. Through LMM analysis, the best prediction models and predictor variables were identified. This LMM framework can easily be extended to other infectious diseases to improve prediction performance and identify important factors affecting the spread of disease.

Several further analyses could be considered in future studies. Firstly, more recent AI/machine learning models such as NARANN and ANFIS, and various ensemble models^34^ could be considered. Secondly, stratified analyses can be conducted. For example, each country can be categorized into low-, middle-, and high-income countries according to their GDP. Such a stratified analysis based on GDP level is expected to provide more accurate prediction results. Lastly, a different strategy for selecting the best hyperparameters could be employed to achieve better results from each model. For example, the best ARIMA model can be selected based on minimum MAPE and RMSE values instead of AIC or BIC^35^.

In this study, different prediction models, vaccination coefficients, and Omicron variants are selected to predict the number of COVID-19 confirmed cases, deaths, and ICU patients for two periods: before Omicron variant and after Omicron variant emergence. Then LMMs were fit using prediction error as response variables and models with the least BIC were selected. Finally, predictor levels that significantly contributed to reducing forecasting error were presented. Lagging was applied to SI and vaccination coefficients of each country since their effects may not be observed immediately. We both predicted using raw and smoothed data. Fitting our LMMs and choosing the best models, we proved that prediction models and Omicron data are significant predictor variables in deciding forecasting accuracies various countries. For the test period before Omicron, the model which uses the prediction model as its only predictor is selected as the best model. Specifically, the ARIMA model shows reasonable performance overall. For the test period after Omicron, the model fitted with only Omicron data was selected as the best model for confirmed cases and raw deaths. For ICU patients and smoothed deaths, the model fitted with only the prediction model was selected as the best model. This indicates that not only do simpler models produce better fitting results but also the choice of the prediction model and Omicron variant data usage are crucial in the improvement of prediction accuracies.

## Methods

### Overview

**Figure 3:**
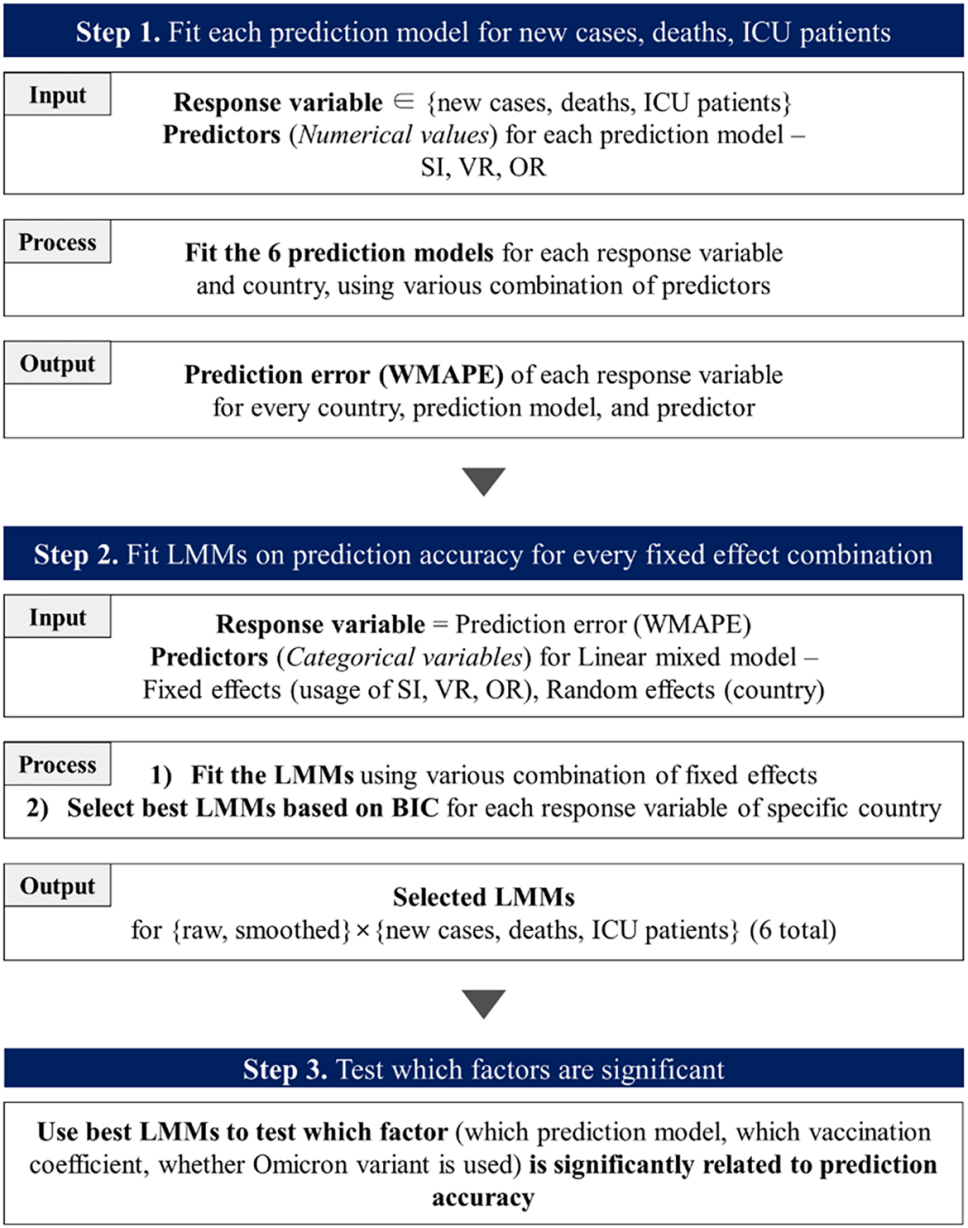
An overview of the study.

Figure 3 is an overview of our study. We divided our prediction periods into before and after Omicron variant emergence and set the last week of each period as test data. For a given country and response variable, we fit each prediction model using VR, SI, and OR (if exists) and obtained Weighted Mean Absolute Percentage Error (WMAPE). Then, using the obtained WMAPE values as response variables and the country as random effects, we fit Linear Mixed Models (LMMs). Fixed effects for our LMMs are the vaccination coefficient, the selection of prediction models, and the Omicron variant (if exists). Note that the predictor variables used in Step 1 (obtaining WMAPE values for each prediction model and country) are numerical values, whereas the predictors used in Step 2 (fitting and finding best LMMs) are categorical variables. In other words, we are primarily interested in determining whether using vaccination coefficient, selection of prediction models, and Omicron variant is significant or not in forecasting. Best LMMs are selected in reverse order of their Bayesian Information Criterion (BIC) and used for hypothesis testing to further determine factors that contribute to reducing prediction error. In short, compared to numerous previous studies that simply concentrated on finding models and variables which will explain given data, we suggest a new approach by first selecting predictors which bring a significant difference in prediction accuracy utilizing LMM and *then* delving into the specific features of each predictor.

### Data collection

The merged data consists of a series of daily confirmed cases, death cases, ICU patients, VRs according to the number of inoculations (per hundred people), and the Stringency Index (SI). All data was downloaded from Our World in Data (OWID)^36^. The daily confirmed cases and deaths were originally collected by the Center for Systems Science and Engineering (CSSE) at Johns Hopkins University^37^, where ICU patient data was officially collected by the OWID team. The SI, which is the overall measure of the strictness of the government’s response to COVID-19, was provided by the Oxford COVID-19 Government Response Tracker^38^. The VR data was collected by Our World in Data group^39^. In addition to this merged data, Korea's ICU data was downloaded from Korea's COVID-19 Dashboard since it was not provided by OWID^40^. Lastly, the proportion of sequences from each country by variant (including Omicron) was downloaded from the CoVariants^41^ and GISAID^42–44^.

### Data preparation

Data from 7 countries (Denmark, United Kingdom, Israel, Japan, South Korea, Singapore, and the United States) were selected for prediction analysis. Data from November 1, 2020, to November 8, 2021, were used for predictions before the Omicron appearance. The last 7 days (November 2, 2021, to November 8, 2021) were used as test data, and the rest were used to train forecasting models. Then data from November 9, 2021, to December 31, 2021, were used for predictions after the Omicron appearance. Similarly, the last 7 days (December 25, 2021, to December 31, 2021) were used as test data, and the rest were used as train data.

We applied the following procedures to handle missing values. First, the missing values preceding the first observation of each variable were all treated as 0. Second, the missing values after the last observation were imputed using the last observation carried forward (LOCF) method, which replaces the missing values at a later time point as the last non-missing observation^45^. Finally, when a missing value exists between actual observations, linear interpolation was used to fill it. The processing of the missing values used the *na.fill()* function and the *na.approx()* function of the R package *zoo*.

As for the test periods, since the VR, SI, and OR were not known yet at the point of prediction, the actual rates, and SI should not be used in prediction. We, therefore, used simulated data (X_t_) for the test period using observed data (X_T-6_ ~ X_T_) of our training period, assuming that rates and SI for the test periods (#1: November 2, 2021, to November 8, 2021; #2: December 25, 2021, to December 31, 2021) will change similar to the last week of the training period (#1: October 26, 2021, to November 1, 2021; #2: December 18 to December 24), to avoid using test data. When the last time point for the train data is T, the simulated data for time *t* was obtained as follows:

$${{\text{X}}}_{{\text{t}}}={{\text{X}}}_{{\text{T}}}+ \frac{(t-T)\sum_{i=T-6}^{T}{X}_{i}}{7}$$

In addition, we decided to use both raw and smoothed data to capture the changing trend and obtain higher prediction accuracy. In our study, smoothed data refers to a 7-day simple moving average since the number of confirmed cases has a weekly seasonal pattern in most countries. This is because the number of diagnostic tests greatly varies over the week. Fewer people are confirmed during weekends than during weekdays since fewer diagnostic tests are carried out during the weekends. Using raw and smoothed data may lead to different parameters for each model, different predictions for each country, and different forecasting accuracies. It is known from several studies^46,47^ that prediction errors from using smoothed data are smaller compared to when using raw data. For example, in stock trading, when price data is smoothed, trends may not be detected on time but detected after a particular time lag. However, there are fewer *error* trades^48^. In this regard, we can assume that our models can better catch the changing trend of the test data, i.e., an abrupt increase or decrease when we use raw data. In comparison, the accuracy itself will be relatively lower when we use smoothed data. Since both have some implications for predicting future data, we examined both data.

### Lagging

The effects of vaccination and intervention policies on the spread of COVID-19 may take some time to be observed. Therefore, it would be reasonable to consider these effects when predicting future daily confirmed cases, daily death cases, or ICU patients. We used a total of 4 lags of 7, 14, 21, and 28 days for VRs and SIs in our models as follows:

$${{\text{Y}}}_{{\text{t}}} \sim {{\text{X}}}_{{\text{t}}-7}+{{\text{X}}}_{{\text{t}}-14}+{{\text{X}}}_{{\text{t}}-21}+{{\text{X}}}_{{\text{t}}-28}$$

where ${{\text{Y}}}_{{\text{t}}}$ is the response variable at time t, and X is the predictor variable with 4 lags.

### Statistical analysis

In this study, statistical models and AI/machine learning models were developed following the previous comparison study^32^, and the mathematical model was developed similarly to the prior mathematical model development research^33^. A brief introduction to forecasting models is provided here, and detailed information can be found in the above mentioned studies and additional references.

### Multiplicative Seasonal ARIMA

AutoRegressive Moving Average (ARMA) models for time series analysis were first suggested in *Time Series Analysis: Forecasting and Control*^49^. Since ARMA models could be applied only to stationary time series, AutoRegressive Integrated Moving Average (ARIMA) models utilize differencing to deal with non-stationary data. Furthermore, multiplicative seasonal ARIMA models were developed to include seasonality in ARIMA models^50^. Unlike prior automatic selections via *auto.arima()*, Akaike Information Criterion (AIC) and Bayesian Information Criterion (BIC) of all potential models were considered to select the best ARIMA model in this study. The selection process identifies the optimal model by pre-setting model orders to integer values, thus preventing overfitting. The *forecast* package in R facilitated fitting, with SI, OR, and VR as additional predictor variables.

### Generalized additive model

The Generalized Additive Model (GAM) is a regression model that captures non-linear relationships between predictor variables and response variables, using smoothing functions^51^. It is assumed that the count time series at a specific time point follows a Poisson distribution, with the logarithmic function serving as the link function. Different smoothing functions were used depending on the predictor variables including cubic splines for weekdays, P-splines for dates, and thin plate regression splines for VR, OR, and SI^52^, respectively. R package *mgcv* was used for fitting GAM models^53,54^.

### TSGLM poisson

Time Series following Generalized Linear Models (TSGLMs) are introduced in *tscount: An R Package for Analysis of Count Time Series Following Generalized Linear Models*^55^. This model analyzes count time series with covariates and history to predict the conditional mean of the series, drawing on past observations and relevant covariates. VR, OR, and SI were considered as covariates in this study. The 'tscount' package in R was utilized for TSGLM Poisson model fitting^55^.

### LightGBM

LightGBM, a gradient boosting decision tree algorithm for regression and classification, iteratively combines weak learners to form a strong model^56^. It employs gradient descent to minimize the loss function, adding models in a greedy manner. The past observation was used as predictor variable after differencing, and VR, OR, and SI were used as covariates in this study. The ‘LightGBM’ package in Python was utilized to construct the model ^56^.

### BiLSTM

To address time series data, Long Short-Term Memory (LSTM) networks is considered as a deep learning approach^57^. Recognizing that LSTM networks process only past information during training, Bidirectional LSTM (BiLSTM) networks were introduced to incorporate backward propagation information as well^58^. In this study, past observation and other covariates (VR, OR, and SI) were used as predictor of this model. In addition, several hyperparameters (bandwidth, layer number, dropout rate) were optimized during the training process. The model was developed in Python version 3.7.6 using *Keras* (Version 2.4.3, https://github.com/keras-team/keras) and *TensorFlow* (Version 2.3.0, https://github.com/tensorflow/tensorflow) libraries.

### Extended SEIR (SEIQRDVP and SEIQRDV3P) model

Mathematical methods can also be used for the prediction of COVID-19 transmission^59–62^. The SEIQRDVP and SEIQRDV3P models, extended versions of the SEIR model, were employed to incorporate the vaccination effect^33^. In the SEIQRDVP model, the vaccinated people still can be infected through contact with the infected group, but with a lower transmission rate. Furthermore, the SEIQRDV3P model differentiates the vaccination group with three vaccination stages (initial, full, and booster dose) to give them with distinct protective efficacies. In addition, the estimation of transmission rate was conducted for distinct periods, delineated by changes in government policy as tracked by the OxCGRT SI. Consequently, SI information was consistently utilized to segment the periods for transmission rate estimation, ensuring its incorporation into the models. The detailed model structure and parameters can be found in our previous study^33^. The fitting process was performed using the Runge–Kutta fourth-order method and *lsqcurvefit* toolbox in MATLAB.

### Forecasting accuracy measures

There are many error measures such as Mean Squared Error (MSE), Mean Absolute Error (MAE), or Mean Percentage Error (MAPE), but each has its drawbacks when used in our analysis. First, since MSE and MAE are scale-dependent measures, they are not suitable for analyzing the prediction performance of different countries. Unlike these, MAPE is a scale-free measure, but it may cause singularity problems when the denominator is zero or can exaggerate the error if the denominator is too small. In this study, we used Weighted Mean Absolute Percentage Error (WMAPE) to measure forecast accuracies for both train and test data. WMAPE is defined as follows:

$${\text{WMAPE}}\, = \,\frac{{\sum \left| {A_{t} - F_{t} } \right|}}{{\sum \left| {A_{t} } \right|}}$$

where A_t_ and F_t_ are actual and forecast values, respectively. The use of WMAPE guarantees the scale-free comparison of forecasting performance between different countries. Also, for convenience in visualization and analysis, we used log_10_WMAPE values as our accuracy measure.

### Linear mixed effects models

In this study, we used LMMs to determine which predictor has the most significant impact on forecast accuracy between prediction models, vaccination covariates, and the use of the omicron variant variable. Generally, LMMs help model correlated multilevel data. We may decide each coefficient to be fixed or random; fixed effects are controlled variables that we are interested in, whereas random effects are considered to be sampled from a population. In other words, fixed effects should show constant coefficients regardless of random effects. In matrix notation, linear mixed models are defined as:

$$y\, = \,X\beta \, + \,Zu\, + \,\varepsilon ,u\,\sim \,N\left( {0, \, G} \right), \, \varepsilon \,\sim \,N(0, \, R)$$

where *β* represents all fixed effects, *u* represents the random effects. X and Z represent design matrices for fixed and random effects, respectively. Note that both random effects and errors follow normal distributions. We used the *lmerTest* package in R for hypothesis testing ^63^. Furthermore, we selected the best LMMs for each indicator using the least Bayesian Information Criterion (BIC) values. Akaike Information Criterion (AIC) ^64^ and BIC are defined as follows:

$$AIC\, = \,2 k{-}2\log (\hat{L})$$

$$BIC\, = \,k\log (n){-}2\log (\hat{L})$$

where *k* is the number of parameters estimated by the model, *n* is the number of observations and $\widehat{L}$ is the maximum likelihood of the model. As AIC and BIC values increase because of variations in the dependent variable and the number of explanatory variables, models with lower AIC and BIC values are preferred. By utilizing LMMs, we can determine the *choice* of which predictor is significant in forecasting each of the given COVID-19 indicators (for both raw and smoothed data). Moreover, after deciding the best LMMs, we performed hypothesis testing on each of the selected models to further determine specific predictor levels that contribute to reducing test error. For example, if our best LMM for confirmed cases suggests that the choice of the prediction model is significant in determining forecasting accuracy, then we proceed to test the impact of each factor (models introduced in Sect. 2.4 and predictor variables) and find which model is significant in reducing test error.

### Supplementary Information

Supplementary Information.

## Data Availability

The datasets used and analyzed during this study are available from the corresponding author on reasonable request.
