# Supplementary material for: Forecasting the spread of COVID-19 based on policy, vaccination, and Omicron data
Source: Sci Rep. 2024 Apr 30;14:9962. doi: 10.1038/s41598-024-58835-9 (PMC11063074; doi:10.1038/s41598-024-58835-9)
Supplement: Supplementary file 1 — Supplementary Information. [file 41598_2024_58835_MOESM1_ESM.docx]

**Supplementary Information**

**Forecasting the spread of COVID-19 based on policy, vaccination, and Omicron data**

Kyulhee Han^1¶^, Bogyeom Lee^2¶^, Doeun Lee^1^, Gyujin Heo^1^, Jooha Oh^3^, Seoyoung Lee^4^, Catherine Apio^1^, Taesung Park^3,*^

1. Interdisciplinary program of bioinformatics, Seoul National University, Seoul, Republic of Korea. 2 Department of Industrial Engineering, Seoul National University, Seoul, South Korea. 3. Department of statistics, Seoul National University, Seoul National University, Seoul, South Korea. 4. College of Liberal Studies, Seoul National University, Seoul, South Korea.

^*^ Corresponding author

E-mail: [tspark@stats.snu.ac.kr](mailto:tspark@stats.snu.ac.kr).

^¶^ These authors contributed equally to this work

**Additional files**

**
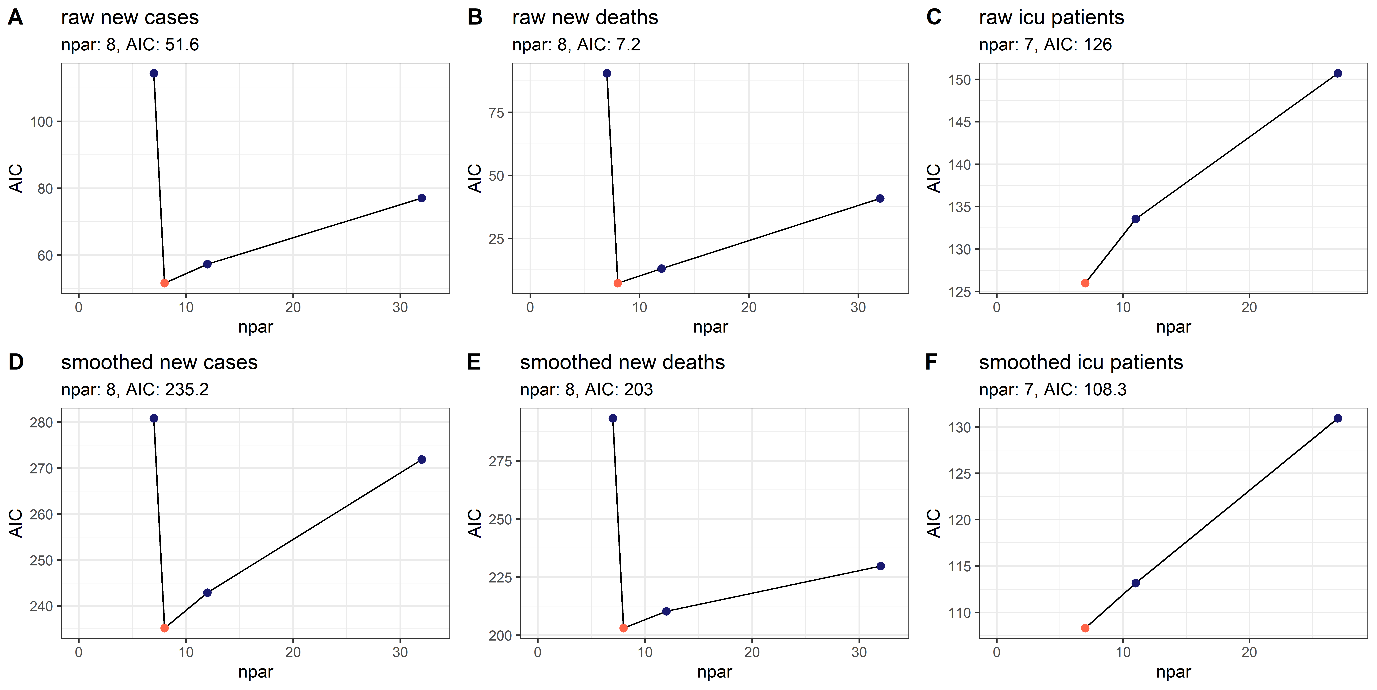
**

**Supplementary Fig. 1.** Visualization of AIC values for each predictor (test period #1).

**
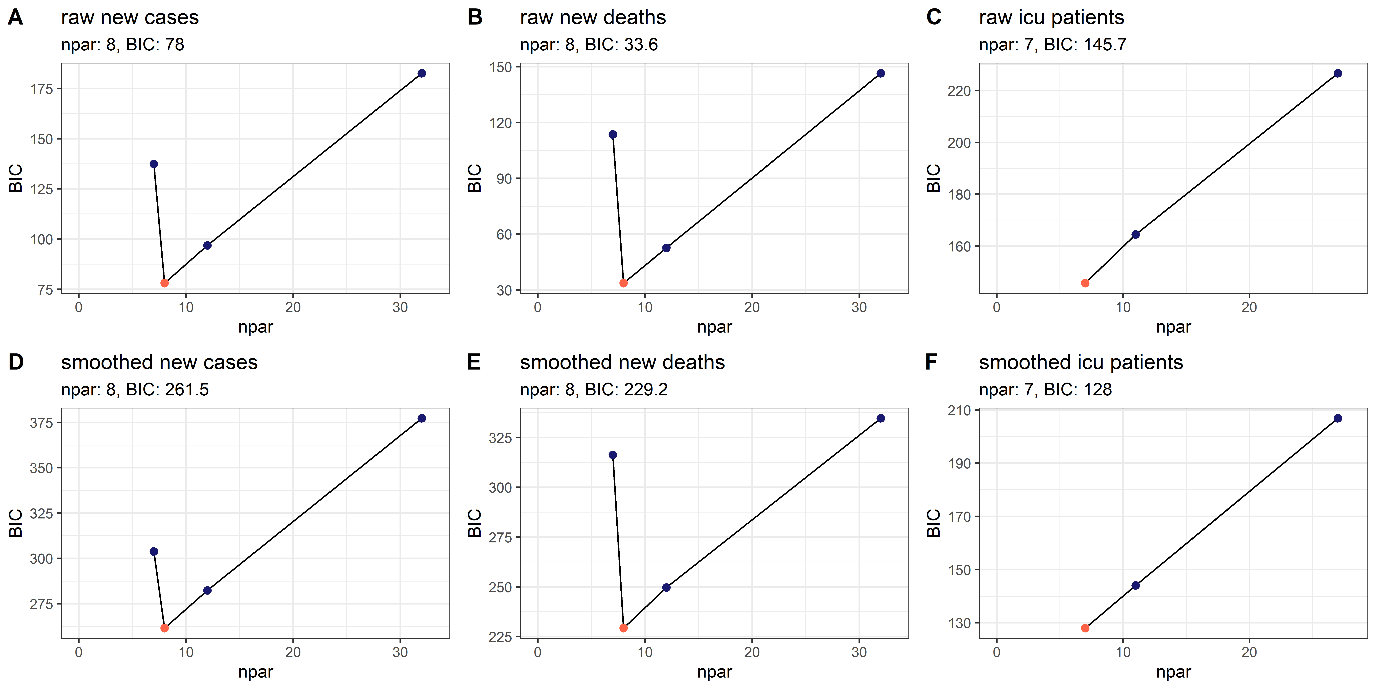
**

**Supplementary Fig. 2.** Visualization of BIC values for each predictor (test period #1).

**
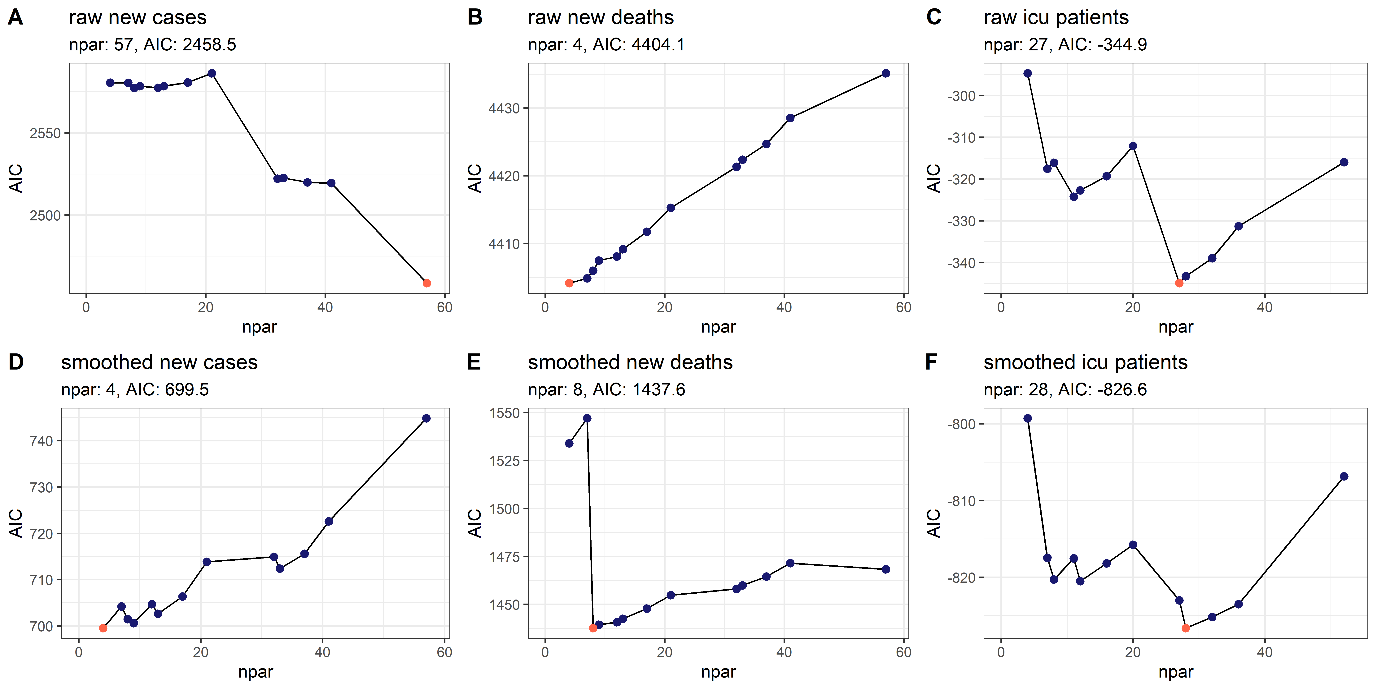
**

**Supplementary Fig. 3.** Visualization of AIC values for each predictor (test period #2).

**
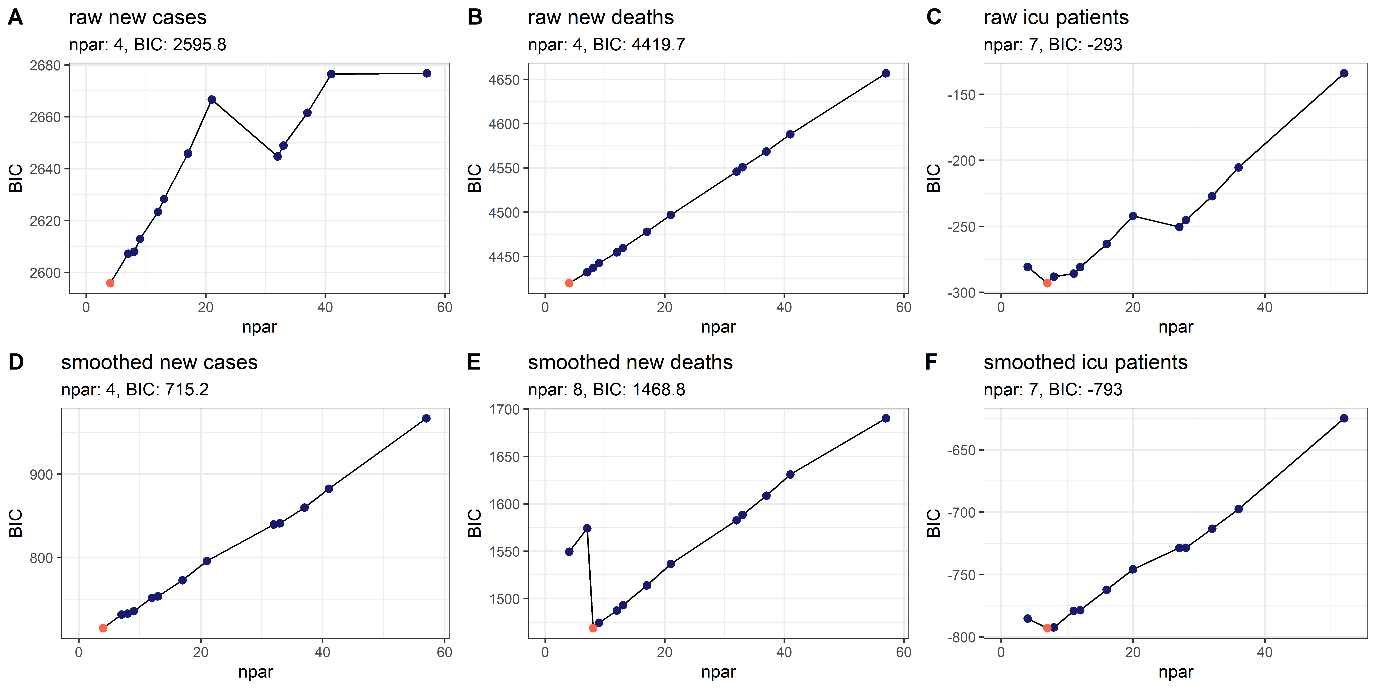
**

**Supplementary Fig. 4.** Visualization of BIC values for each predictor (test period #2).
